# *Sicyos angulatus* Prevents High-Fat Diet-Induced Obesity and Insulin Resistance in Mice

**DOI:** 10.7150/ijms.42247

**Published:** 2020-03-05

**Authors:** Ji Hyun Choi, Jung-Ran Noh, Yong-Hoon Kim, Jae-Hoon Kim, Eun-Jung Kang, Dong-Hee Choi, Jung Hyeon Choi, Jin-Pyo An, Won-Keun Oh, Chul-Ho Lee

**Affiliations:** 1 Laboratory Animal Resource Center, Korea Research Institute of Bioscience and Biotechnology (KRIBB), 125 Gwahak-ro, Yuseong-gu, Daejeon 34141, Republic of Korea; 2 Department of Functional Genomics, KRIBB School of Bioscience, University of Science and Technology (UST), 217 Gajeong-ro, Yuseong-gu, Daejeon, Korea; 3 Korea Bioactive Natural Material Bank, Research Institute of Pharmaceutical Sciences, College of Pharmacy, Seoul National University, 1 Gwanak-ro, Gwanak-gu, Seoul 08826, Republic of Korea

**Keywords:** * Sicyos angulatus*, high-fat diet, obesity, lipogenesis, insulin resistance

## Abstract

Obesity is a medical condition in which excess body fat has accumulated to a serious extent. It is a chronic disease that can lead to dyslipidemia, insulin resistance, and type 2 diabetes. In the present study, we investigated the anti-obesity effects of *Sicyos angulatus* (SA) extract on a high-fat diet (HFD)-induced C57BL/6J obese mice. The mice were divided into vehicle and three SA groups (25, 50, and 100 mg/kg body weight). The mice were fed a HFD with or without SA for 12 weeks. The oral administration of SA reduced body and adipose tissue weight in HFD-fed mice compared to those in the vehicle group (*p*<0.05). Adipocyte size and inflammation significantly decreased in the SA-administered groups in a dose-dependent manner. In particular, adipocytes larger than 5000 µm^2^ were remarkably reduced by around 50% in the SA-treated groups (*p*<0.05). In addition, SA contributes towards reducing insulin resistance (measured as the HOMA-IR index) and glucose intolerance in HFD-induced obese mice (*p*<0.05; Vehicle 21.5±3.1 vs. SA100 4.7±0.4). These beneficial effects of SA on obesity may be linked to the suppression of lipogenesis and stimulating energy metabolism in white adipose tissue and muscle. In white adipose tissue and muscle, the administration of SA activated AMPK pathway, leading to the inhibition of the development of pathophysiological conditions associated with obesity, including lipogenesis and inflammation. These findings suggest that SA may prevent obesity through inhibiting fat accumulation in HFD-induced obese mice. Therefore, SA is able to exert metabolic benefits in the prevention of obesity and insulin resistance.

## Introduction

Obesity is rapidly increasing in all countries around the world. It is a global epidemic that is consistently recognized as a health problem. According to a recent report, the increase in the world obesity rate from 1975 to 2014 was 3.2-10.8% in men and 6.4-14.4% in women 1.

Obesity is defined as an imbalance between energy intake and energy expenditure. When energy intake exceeds energy expenditure, fat accumulates in the body 2. Expanded adipose tissue is closely related to the creation of an inflammatory environment in conditions, including obesity and its comorbidities 3. Inflammation, induced by excessive adipose tissue accumulation, appears to link obesity to disease risk. Numerous epidemiological studies have demonstrated that the excessive accumulation of visceral adipose tissue is associated with pathological conditions, such as hypertension, dyslipidemia (increased circulating triglycerides, free fatty acids, LDL to HDL ratio) 4, 5, cardiovascular disease, type 2 diabetes 6, 7, and non-alcoholic fatty liver disease 8. Visceral adipose tissue can cause metabolic abnormalities by secreting inflammatory adipokines, such as interleukin (IL)-6, tumor necrosis factor-α (TNFα), macrophage chemoattractant protein-1 (MCP-1), and resistin, which induces insulin resistance and diabetes 9, 10. Currently, phentermine, orlistat, and liraglutide are the drugs used for obesity and insulin resistance, but there are some side effects, such as headaches, abdominal pain, insomnia, and nausea 11. Therefore, despite the present challenges, the development of novel, safe therapeutics to prevent and treat obesity are urgently required.

*Sicyos angulatus* (SA) is an annual plant that belongs to the gourd family Cucurbitaceae, which originated from North America 12, 13. SA is spread throughout Asia as well as Europe, and because of its small, hard spines and high fertility, it inhibits the growth of other plants 14. In particular, the Korean Ministry of Environment designated its thorns as an ecological disturbance. However, before flowering, the young leaf of this plant is listed as an edible raw material in the Korean Food Standard Codex (KFSC), and thus the possibility of SA being a functional food has been suggested. The biological and potential therapeutic effects of SA in acute liver injury and atherosclerosis mouse model have been reported 15, 16. More recently, functions of SA in liver were demonstrated through *in vitro* and *in vivo* experiment. The inhibitory effect of flavone glycosides from SA extract on hepatic lipid accumulation induced by high concentrations of palmitic acid and glucose in HepG2 cells was reported 17. Also, anti-hepatic steatosis activities of SA and its active compound kaempferol 3-O-[α-L-rhamnopyranosyl-(1→6)]-β-D-glucopyranosyl-7-O-α-L-rhamnopyranoside were demonstrated in high-fat diet (HFD)-induced obese model 18. This effect was probably mediated by suppressing the hepatic lipid accumulation and regulating lipogenic gene expression in the liver. Based on these results, we expect SA to be effective against obesity, so the study focused on adipose tissue and muscle. The aim of this study was to investigate the effects of SA on obesity and insulin resistance in HFD-induced obesity mouse models.

## Materials and Methods

### Plant collection and SA extract preparation

The collection and extract preparation of SA were performed by Korea Bioactive Natural Material Bank (KBNMB) in Korea. In brief, the leaves and stems of SA were collected from the Medicinal Plant Garden of the College of Pharmacy, Seoul National University, Goyang-si. Gyeonggi-do, Korea, (37° 71' 27"N, 126° 81' 88"E). A voucher specimen (SNUPMHG-014271) was deposited in the Herbarium of the Medicinal Plant Garden of the College of Pharmacy, Seoul National University. The dried stems and leaves of SA (5 kg) were extracted twice (8 h × 2) with 70% ethanol (50 L) under reflux conditions at 80 °C. The extract was concentrated under reduced pressure until dried. The residue was frozen at -80 °C and lyophilized to yield dried powder for animal experiments.

### Animals

Male, eight-week-old C57BL/6J mice were bred and maintained at the Korea Research Institute of Bioscience and Biotechnology (Daejeon, Republic of Korea). The mice were housed in plastic cages in a temperature-controlled (22 ± 1°C) facility and were maintained on a reverse 12 h light/dark cycle. For the animal experiments, SA was dissolved in 0.5% carboxymethyl cellulose (CMC), and the mice were randomly divided into four groups (n=5/group): high-fat diet (HFD, catalog number 12492; Research diets Inc., Bethlehem, PA, USA) plus vehicle and HFD plus three different doses of SA (25, 50, and 100 mg/kg). Dosage of SA used in this study was determined by preliminary tests and there was no toxicity at the concentration administered. SA were daily administered at a certain time by oral gavage for 12 weeks. Mice were weighed every week, and fasting blood glucose levels were measured at the end of the experiment. All animal experiments were approved by the Institutional Animal Care and Use Committee and performed in accordance with the institutional guidelines of the Korea Research Institute of Bioscience and Biotechnology.

### Plasma lipid analysis

At the end of the experimental period, mice were fasted overnight and blood samples were taken from the orbital venous congestion to determine the concentrations of plasma biomarkers. Plasma samples were prepared by centrifugation of the blood samples at 10,000 rpm for 5 min at 4°C, and the samples were stored at -70°C until analysis. The plasma triglyceride and total cholesterol levels were measured using an automatic blood chemistry analyzer (AU480; Beckman Coulter, Krefeld, Germany).

### Plasma cytokine and leptin measurement

The levels of plasma cytokines (TNF-α, IL-6 and IL-1β) and leptin were measured via cytokine OptEIA™ kit (BD Biosciences) and leptin ELISA kit (R&D System), respectively, according to the manufacturers' instructions. The optical density was determined using a microplate reader set to 450 nm.

### White adipose tissue analysis

The white adipose tissue, epididymal and inguinal fat were removed from the mice and fixed in 10% neutral buffered formalin, then embedded in paraffin and cut into 6 μm-thick sections. Some sections were stained with hematoxylin and eosin (H&E) for microscopic measurements of cell sizes. To measure adipocyte diameter using a light microscope at a magnification of 100×, ten random fields per tissue were evaluated with ImageInside software.

### Immunohistochemistry

The other sections were stained with F4/80 antibody to measure macrophage infiltration into the adipose tissue. The paraffin sections were deparaffinized and hydrated using xylene and graded alcohol series. Sections were boiled in citrate buffer using a microwave oven for antigen retrieval. To quench endogenous peroxidase activity, sections were placed for 30 minutes in 30% hydrogen peroxide. After washing with buffer, each section was blocked with normal blocking serum to prevent non-specific binding of the antibody. After removing the blocking solution, the primary antibody was diluted to a ratio of 1:100 and was added and incubated overnight at 4 °C in a humidified chamber. The next day, the diluted biotinylated secondary antibody was added. After removing the antibody solution and washing the sections, the VECTASTAIN Elite ABC Reagent was applied. Finally, the samples were visualized using 3,3'-diaminobenzidine.

### Crown-like structure (CLS) quantification

CLSs were identified as clusters of macrophages that had infiltrated the adipose tissue and formed ring-like structures. The CLS images were quantified by imaging with a microscope at a magnification of 200 ×. The number of CLSs was manually counted in five random fields for each section. Counting the CLSs was determined using ImageJ software from IHC-stained images of epididymal fat.

### Glucose tolerance test and insulin tolerance test

After ten weeks of HFD treatment with SA administration, intraperitoneal glucose tolerance test (IP-GTT) and intraperitoneal insulin tolerance test (IP-ITT) were performed. After 16 or 4 h of fasting for GTT and ITT 19, respectively, the basal glucose level (0 min) of each mouse was measured from blood taken from the tail vein. Then, either glucose (2 g/kg) or insulin (0.1 U/ml) was injected intraperitoneally, and blood glucose levels were monitored at 30, 60, 90, and 120 min. The area under the curve (AUC) was calculated using GraphPad Prism software (La Jolla, CA, United States). Plasma insulin levels were determined using an ELISA kit (Mercodia, Uppsala, Sweden) according to the manufacturer's instructions. The homeostasis model assessment (HOMA) was used to calculate the homeostatic index of insulin resistance (HOMA-IR) as follows: HOMA-IR=[fasting glucose (mmol/L) × fasting insulin (μIU/ mL)]/22.5.

### Reverse transcription-quantitative polymerase chain reaction (RT‑qPCR)

Total RNA was isolated from the mouse adipose tissue and muscle using TRIzol reagent (Invitrogen; Thermo Fisher Scientific, Inc.). Adipose tissues were homogenized in TRIzol reagent with stainless steel beads using a TissueLyser (Qiagen GmbH, Hilden, Germany). Subsequently, cDNA was synthesized from 1 μg of total isolated RNA using a cDNA synthesis kit (iScript™ cDNA synthesis kit; Bio‑Rad Laboratories, Inc., Hercules, CA, USA) according to the manufacturer's protocol. Subsequently, qPCR was performed using SYBR Green PCR MasterMix (AccuPower® 2X Greenstar™ qPCR MasterMix; Bioneer Co., Daejeon, Korea) and the StepOne™ Real-time PCR system (Applied Biosystems; Thermo Fisher Scientific, Inc.). The cycling conditions were as follows: pre‑denaturation at 95 °C for 10 min, followed by denaturation at 95 °C for 10 sec, and annealing and extension at 60 °C for 30 sec for 45 cycles of amplification. All expression data were normalized to 18S ribosomal RNA and were calculated using the 2‑^ΔΔ^Cq method. The results are presented in terms of the fold-change relative to the expression in the vehicle group. The PCR primer pair sequences are detailed in **Table 1**.

### Western blot analysis

Mouse muscle was homogenized in lysis buffer (0.1 mmol/L sodium vanadate, 1 mmol/L phenylmethanesulfonyl fluoride, 25 mmol/L NaF, 50 mmol/L Tris-HCl, 40 mmol/L glycol phosphate, 120 mmol/L NaCl, 1% NP40 and 0.5% Triton X-100) containing protease inhibitor and phosphatase inhibitor. The homogenates were centrifuged at 13,000 rpm for 15 min and the protein concentration in the supernatant was measured using the Bradford method. Protein samples were separated by electrophoresis on 10% a sodium dodecyl sulphated-polyacrylamide gel and transferred to a polyvinylidene fluoride transfer membrane. The membranes were blocked with 5% skim milk for 1 h and incubated with a primary antibody overnight at 4℃. The next day, the membranes were washed three times with Tris-buffered saline-Tween-20 (TBST) and incubated with a HRP-conjugated secondary antibody for 1 h at room temperature. After washing with TBST, bands were detected using EzWestLumi plus (ATTO). Proteins in western blots were quantified by densitometry using TINA software, 2.09; (Raytest Isotopenmessgeräte, Straubenhardt, Germany).

### Statistical analysis

Numerical data are presented as the mean ± standard error of the mean (SEM). JMP 5.1 software was used for analysis (SAS Institute, Inc., Cary, NC, USA). Comparisons between the vehicle- and SA-treated groups were performed using a two‑tailed Student's *t*‑test. A *P* value < 0.05 was considered statistically significant.

## Results

### SA reduces body weight gain and adipose tissue weight in obese mice fed a HFD

**Figure 1:**
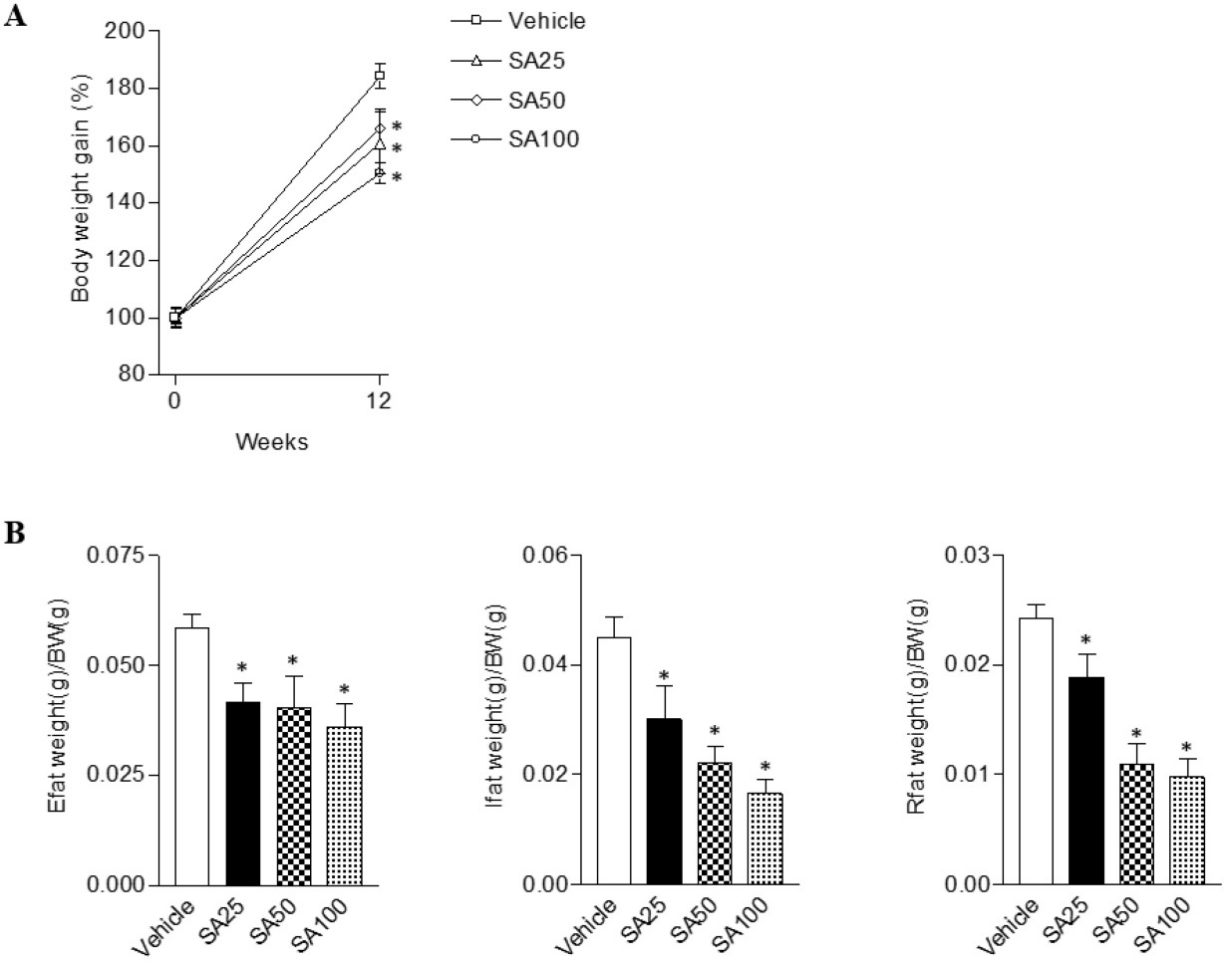
** The effects of SA on body and white adipose tissue weights in C57BL/6 mice fed a high-fat diet.** Mice were randomly divided into four groups: high-fat diet (HFD) plus vehicle (0.5% CMC) and HFD plus SA (25, 50, and 100 mg/kg). The mice were fed a HFD only or HFD with SA for 12 weeks. Body (A) and white adipose tissue (Epididymal, inguinal and retroperitoneal fat) weights (B). Values are the mean±SEM (*n* = 5/group). *Significantly different from the HFD-fed vehicle group at *p* < 0.05.

To evaluate the anti-obesity effects of SA, we analyzed the body and white adipose tissue weights in HFD-induced obese mice treated with SA for 12 weeks. The vehicle group was fed a HFD plus 0.5% CMC and the other three groups were fed HFD plus SA (25, 50, and 100 mg/kg). In the SA-treated groups, body weight gain significantly decreased compared with the vehicle group (SA100 group: 17.2±2.0% decrease) (**Figure 1A**). White adipose tissues (WAT), epididymal, inguinal, and retroperitoneal fat weights were measured to examine whether the reduced body weight gain in the SA treatment groups was associated with decreased body fat accumulation (**Figure 1B**). Epididymal fat weight in the SA25, 50, and 100 groups was dose-dependently decreased by 29%, 31%, and 39%, respectively. Similarly, in the case of the inguinal fat, compared to the vehicle group, weight was significantly reduced by 33% at 25 mg/kg, 50% at 50 mg/kg, and 63% at 100 mg/kg SA treatment groups. In addition, retroperitoneal fat weight also significantly decreased in the SA-treated groups compared with the vehicle group. These results suggest that SA lowers body weight gain by reducing fat weight.

### SA reduces the adipocyte size in epididymal and inguinal fat in obese mice fed a HFD

**Figure 2:**
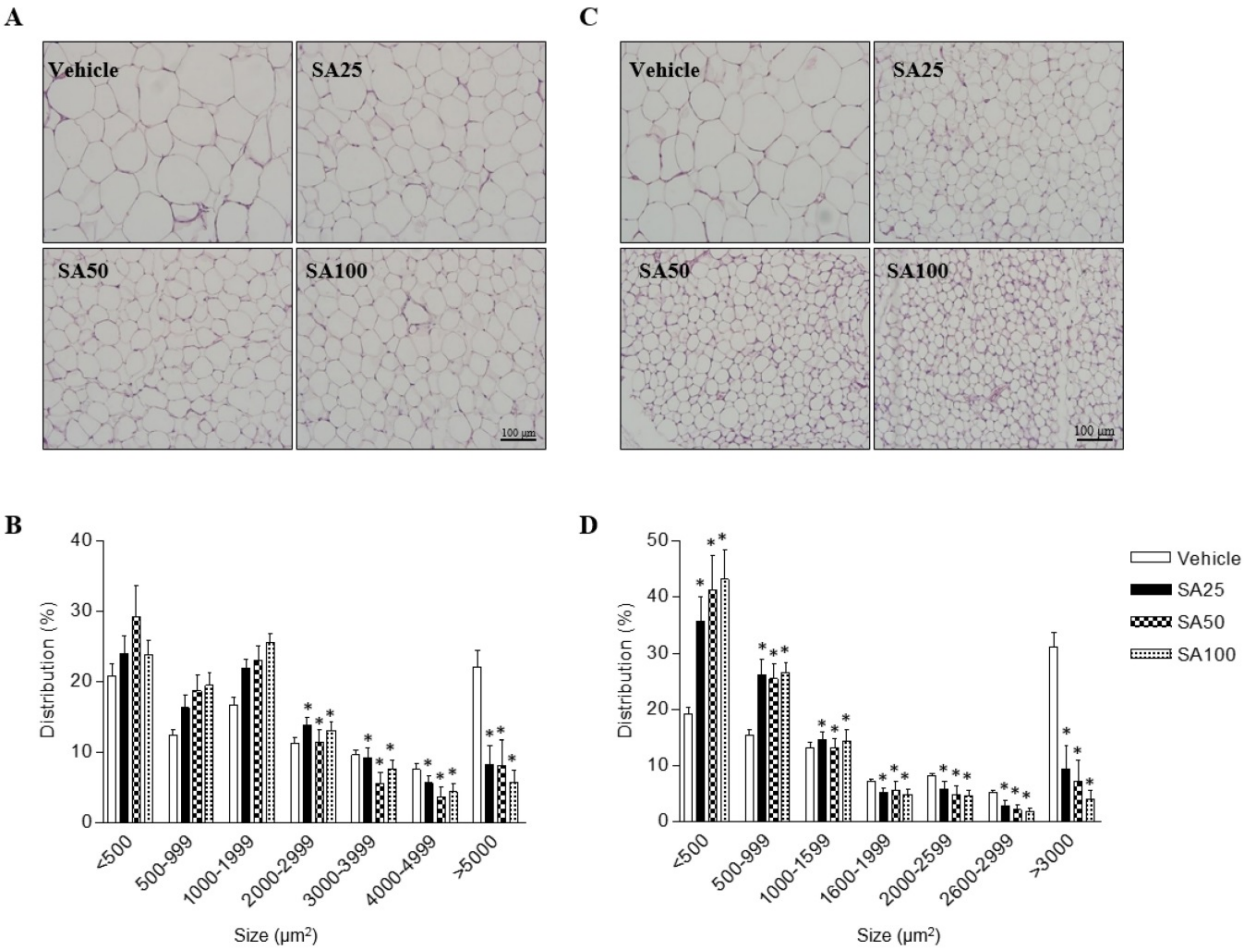
** The effect of SA on adipose tissue cell size distribution in C57BL/6 mice fed a high-fat diet.** Mice were randomly divided into four groups: high-fat diet (HFD) plus vehicle (0.5% CMC) and HFD plus SA (25, 50, and 100 mg/kg). The mice were fed a HFD only or HFD with SA for 12 weeks. Representative H&E-stained images (A and C) and the size distribution of epididymal (B) and inguinal (D) adipose tissue deposits. Values are the mean±SEM (*n* = 5/group). *Significantly different from the HFD-fed vehicle group at *p* < 0.05.

In order to investigate the effects of SA on adipose cell-size distributions, morphological observations of epididymal and inguinal adipocytes were assessed by H&E staining. Adipocyte size in both adipose tissues was shown to be smaller in SA-administered groups than that in the vehicle group, depending on the SA dose (**Figures 2A and C**). The number of adipocytes of less than 2000 μm^2^ in epididymal fat tended to increase in the SA treatment groups compared to that in the vehicle group, whereas those of more than 2000 μm^2^ were significantly reduced. In particular, adipocytes larger than 5000 μm^2^ were remarkably reduced by around 50% in the SA-treated groups (**Figure 2B**)**.** In inguinal fat, adipocytes larger than 1600 μm^2^ were significantly decreased in the SA groups compared with those in the vehicle group. Adipocytes smaller than 500 μm^2^ significantly increased while adipocytes larger than 3000 μm^2^ decreased in the SA-treated groups. Noteworthily, adipocyte distribution over 3000 μm^2^ decreased by 85% in the SA100 group compared with that in the vehicle group (**Figure 2D**). These results suggest the SA prevents the accumulation of fat in WAT in HFD-induced obese mice.

### SA ameliorates the aggravation of adipose tissue inflammation in mice fed a HFD

**Figure 3:**
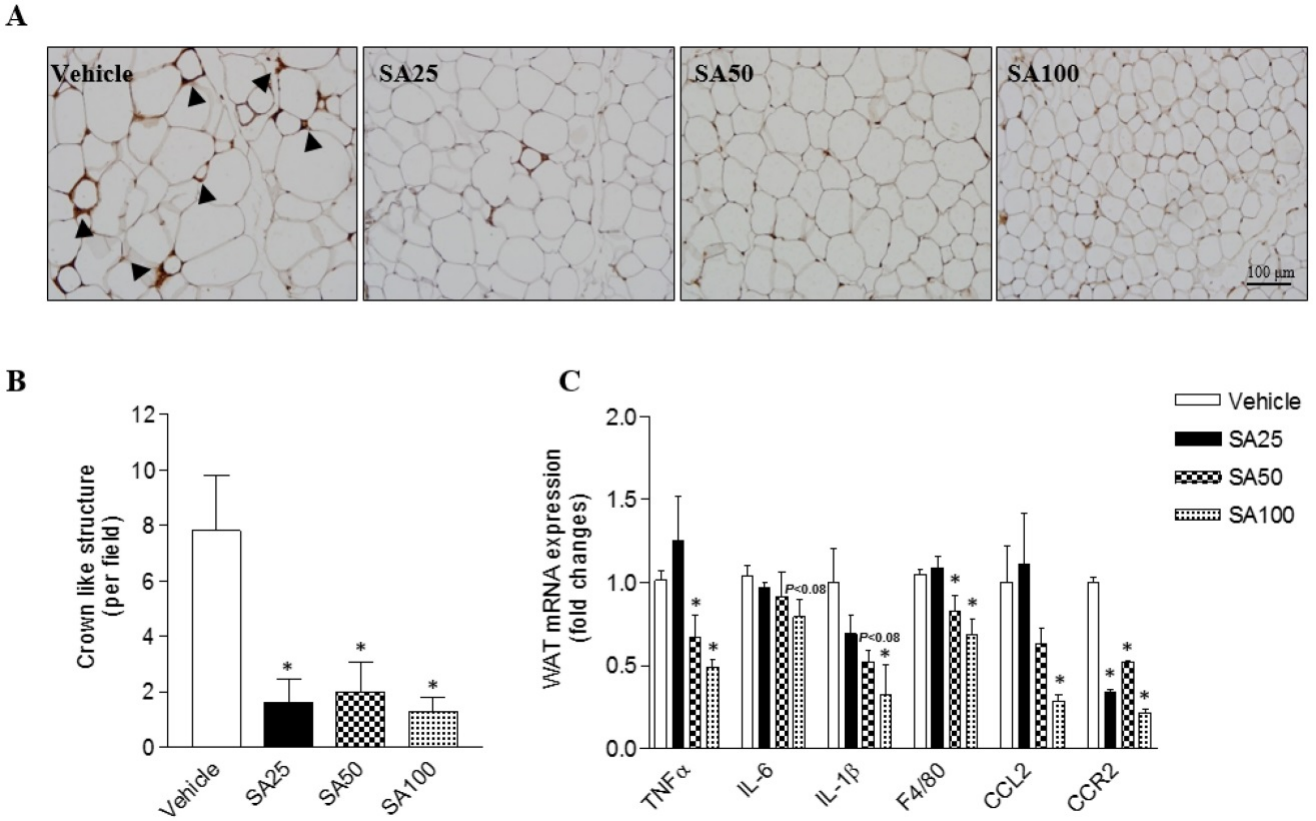
** Effect of SA on adipose tissue inflammation in C57BL/6 mice fed a high-fat diet.** Mice were randomly divided into four groups: high-fat diet (HFD) plus vehicle (0.5% CMC) and HFD plus SA (25, 50, and 100 mg/kg). The mice were fed a HFD only or HFD with SA for 12 weeks. Representative images of epididymal fat stained with an anti-F4/80 antibody (A), quantification of crown-like structures (B), and inflammatory gene expression of epididymal fat (C). A black triangles indicate macrophages surrounding adipocytes. Values are the mean±SEM (*n* = 5/group). *Significantly different from the HFD-fed vehicle group at *p* < 0.05.

Obesity is characterized by the infiltration of macrophages into WAT 20. Therefore, in this study, macrophage infiltration was examined through immunohistochemistry using an F4/80 antibody for the detection of macrophages/monocytes in epididymal fat. Histology revealed a decrease in macrophage infiltration into adipose tissue (**Figure 3A**). Crown like structure (CLS) consist of macrophages surrounding dying or dead adipocytes 21. CLS formation commonly occurred in the HFD-induced obese mice in this study (vehicle group: 7.6±2.0 CLS per field), but significantly decreased by around six-fold in SA groups (1.3-2.0±1.0 CLS per field) (**Figure 3B**). In support of the histology data, the mRNA expression of inflammation-related markers was confirmed. TNFα is an inflammatory cytokine produced by macrophages during inflammation, leading to necrosis or apoptosis 22. The WAT mRNA expression of TNFα significantly decreased in the SA50 and 100 groups. In addition, expression of IL-6, IL-1β, F4/80, CCL2, and CCR2 was markedly reduced in the epididymal fat of SA-treated groups relative to that in the vehicle group (**Figure 3C**). These results suggest that SA administration alleviates the adipose tissue inflammation by suppressing macrophage infiltration and regulating inflammatory gene expression.

### SA attenuates biochemical abnormalities in mice fed a HFD

**Figure 4:**
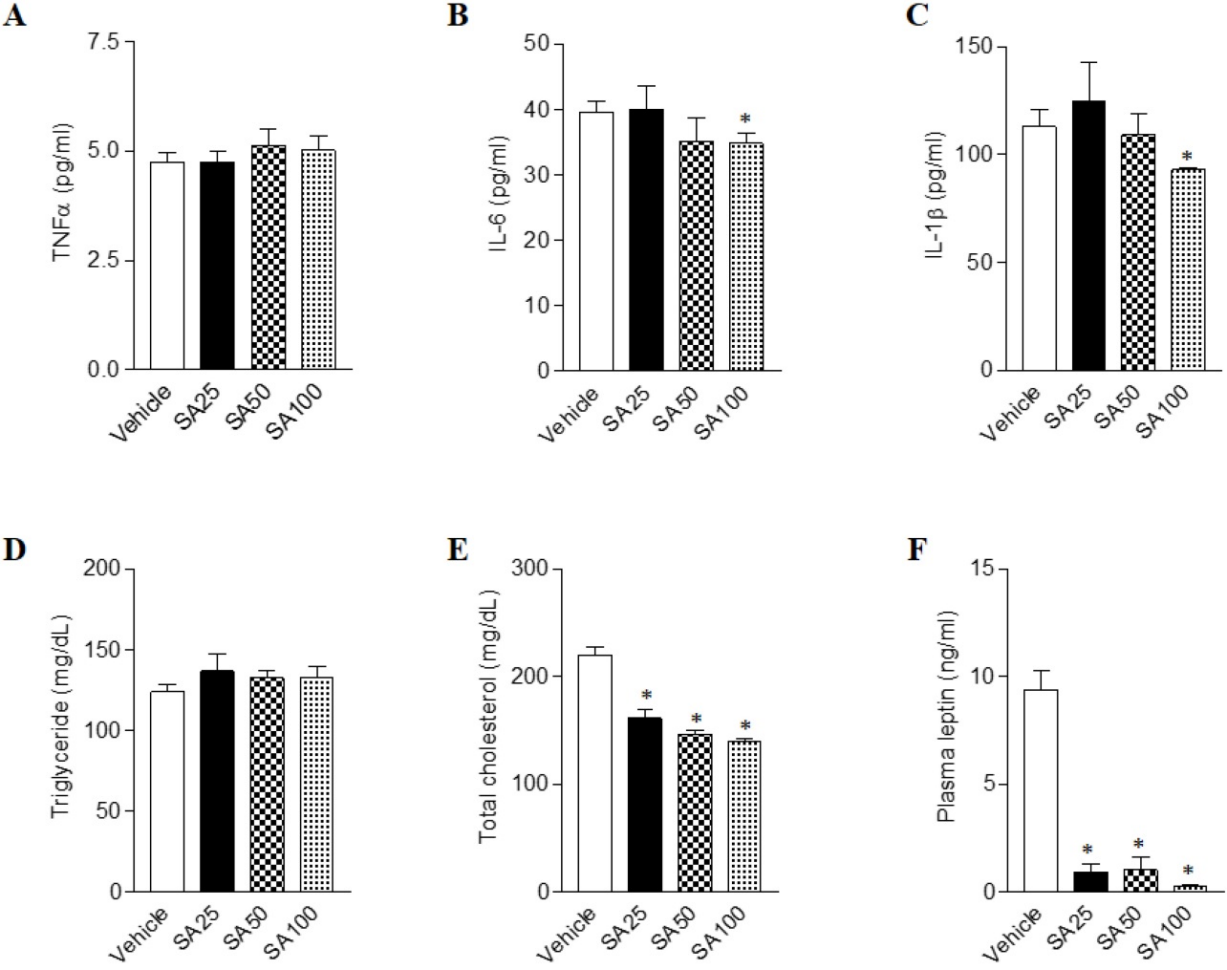
** Effects of SA on plasma cytokines, lipids and leptin levels in C57BL/6 mice fed a high-fat diet.** Mice were randomly divided into four groups: high-fat diet (HFD) plus vehicle (0.5% CMC) and HFD plus SA (25, 50, and 100 mg/kg). The mice were fed a HFD only or HFD with SA for 12 weeks. Plasma cytokines (A-C), lipids (D and E) and leptin (F) levels measured after 12 weeks of treatment. Values are the mean±SEM (*n* = 5/group). *Significantly different from the HFD-fed vehicle group at *p* < 0.05.

Long term HFD results in increased secretion of inflammatory cytokine, leading to chronic inflammatory reactions 23. In order to test whether the amount of inflammatory cytokine in the blood actually decreased by SA treatment, we detected the representative inflammatory cytokines TNFα, IL-6 and IL-1β in plasma (**Figure 4A-C**). We found that IL-6 and IL-1β were significantly decreased in the SA100 groups. Next, the plasma levels of obesity- related biomarkers in SA-treated mice with a HFD were measured. Although plasma triglyceride level showed no significant change upon SA treatment, cholesterol levels significantly decreased in SA groups in a dose-dependent manner (**Figures 4D and E**). Obese mice have high levels of leptin, which is secreted by adipocytes, but the leptin signal does not function normally in these mice due to a condition known as leptin resistance 24. In this study, HFD-fed vehicle group showed high levels of plasma leptin, indicating leptin resistance. However, SA-treated groups showed significantly lower plasma leptin levels compared to the vehicle group (**Figure 4F**). These results support the anti-obesity effects of SA in HFD-induced obese mice.

### SA improves glucose intolerance and insulin resistance in mice fed a HFD

**Figure 5:**
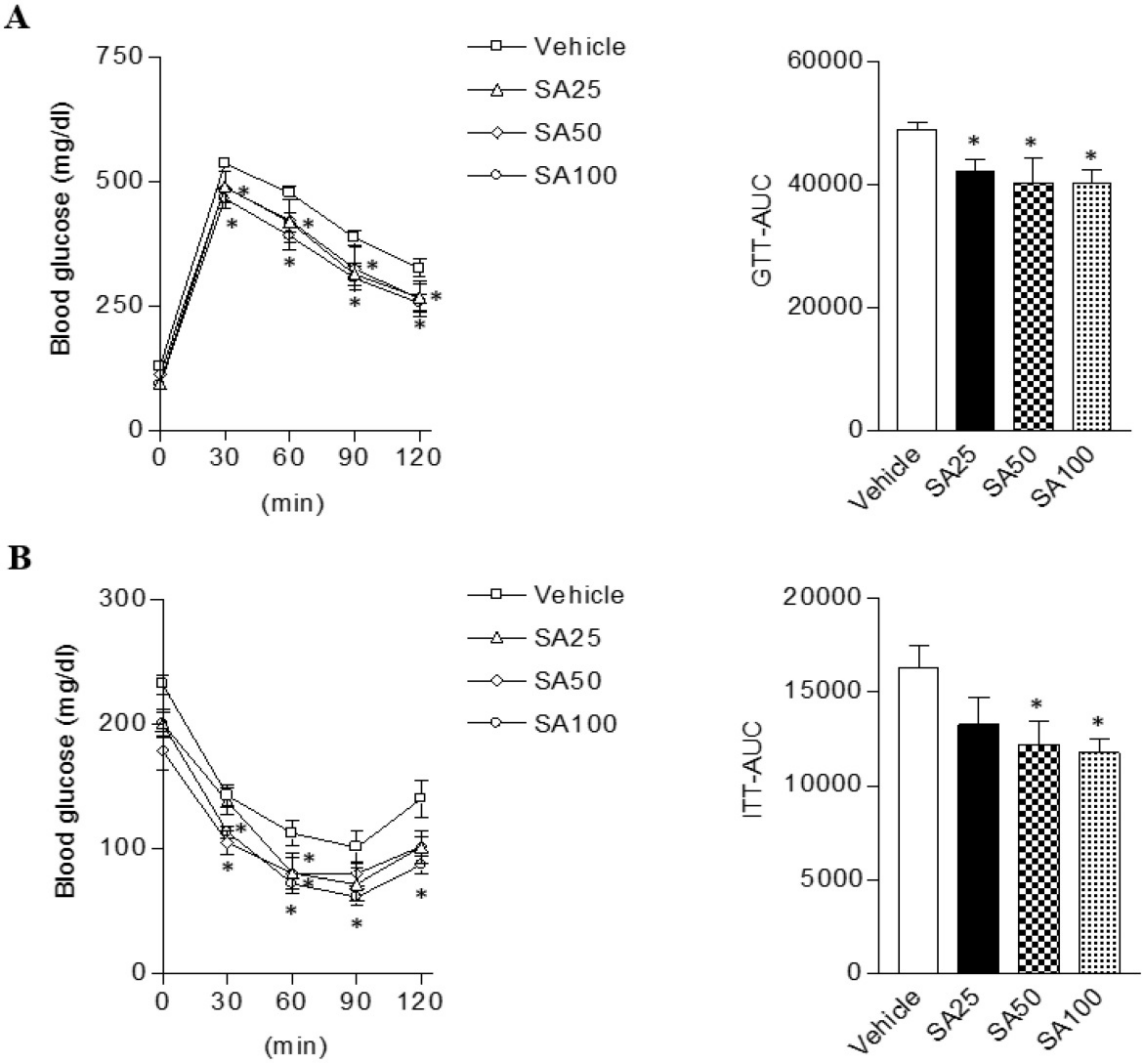
** The effect of SA on glucose and insulin tolerance in C57BL/6 mice fed a high-fat diet.** Mice were randomly divided into four groups: high-fat diet (HFD) plus vehicle (0.5% CMC) and HFD plus SA (25, 50, and 100 mg/kg). The mice were fed a HFD only or HFD with SA for 12 weeks. Blood glucose change and the mean area under the curve (AUC) measured during the IP-GTT (A) and -ITT (B). Values are the mean±SEM (*n* = 5/group). *Significantly different from the HFD-fed vehicle group at *p* < 0.05.

**Figure 6:**
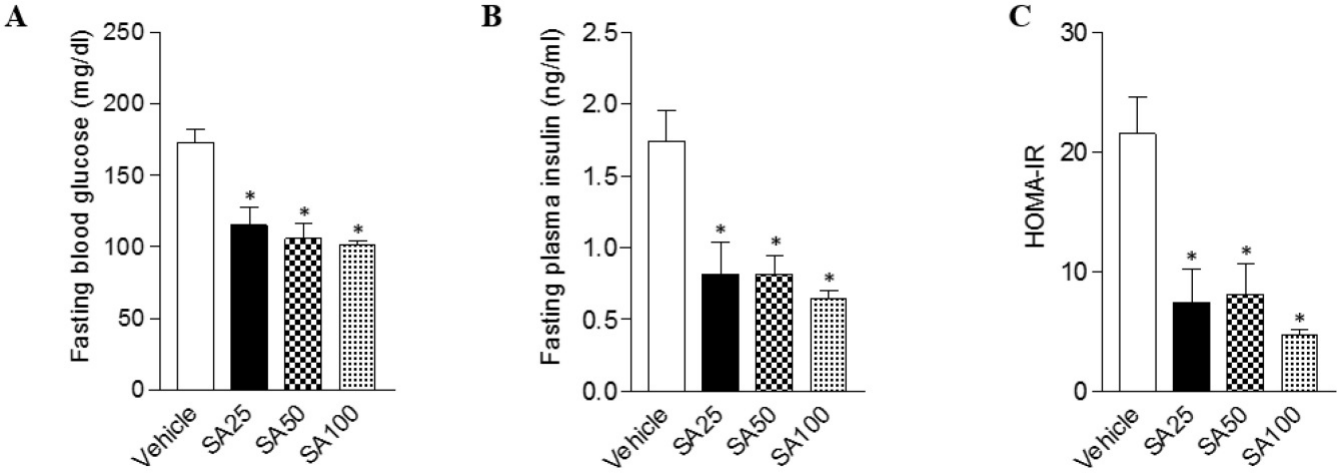
** The effects of SA on fasting blood glucose, insulin, and the HOMA-IR index in C57BL/6 mice fed a high-fat diet.** Mice were randomly divided into four groups: high-fat diet (HFD) plus vehicle (0.5% CMC) and HFD plus SA (25, 50, and 100 mg/kg). The mice were fed a HFD only or HFD with SA for 12 weeks. Fasting blood glucose (A), insulin (B), and the HOMA-IR index (C) measured after 12 weeks of treatment. Values are the mean±SEM (*n* = 5/group). *Significantly different from the HFD-fed vehicle group at *p* < 0.05.

Obesity can cause insulin resistance and type 2 diabetes 25. Therefore, glucose and insulin tolerance was measured ten weeks after SA treatment. When GTT was performed through glucose injection, fasting blood glucose levels were lower in SA-treated groups, and in particular were lowest in the SA100 group (**Figure 5A**). Moreover, the SA-treated groups showed a significant decrease in blood glucose levels after insulin injection at 30 and 60 min. Especially, these levels were consistently suppressed for 120 min after injection with insulin in SA100 group (**Figure 5B**). The area under the curve (AUC) in each experiment's results was also consistent with the blood glucose measurements. GTT-AUC showed a significant decrease of 18% in the both SA50 and SA100 group compared with the vehicle group. In addition, ITT-AUC showed a reduction of 25% in the SA50 group and 28% in the SA100 group. The HOMA-IR is used to measure the severity of insulin resistance 26. When HOMA-IR was calculated using fasting blood glucose and insulin levels measured at 12 weeks (**Figure 6A-C**), the SA-treated groups (SA100 group: 4.7±0.4) showed a significantly lower value than the vehicle group (21.5±3.1), suggesting that insulin resistance was improved by SA treatment.

### SA regulates the gene expression of lipid and energy metabolism in WAT and muscle of mice fed a HFD

**Figure 7:**
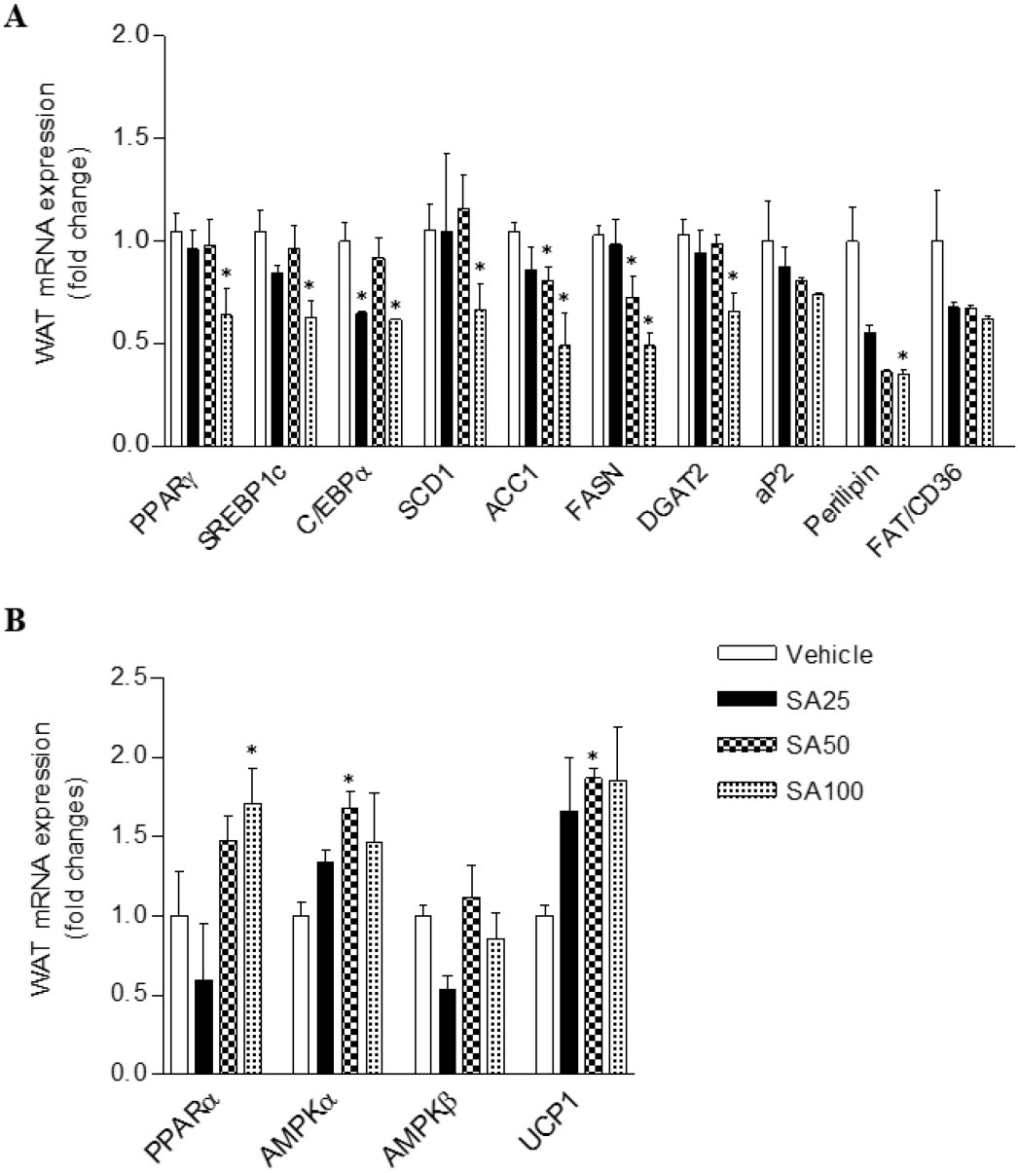
** The effect of SA on the mRNA expression of white adipose tissue lipids (A) and energy metabolism (B) genes in C57BL/6 mice fed a high-fat diet.** Mice were randomly divided into four groups: high-fat diet (HFD) plus vehicle (0.5% CMC) and HFD plus SA (25, 50, and 100 mg/kg). The mice were fed a HFD only or HFD with SA for 12 weeks. Values are the mean±SEM (*n* = 5/group). *Significantly different from the HFD-fed vehicle group at *p* < 0.05.

**Figure 8:**
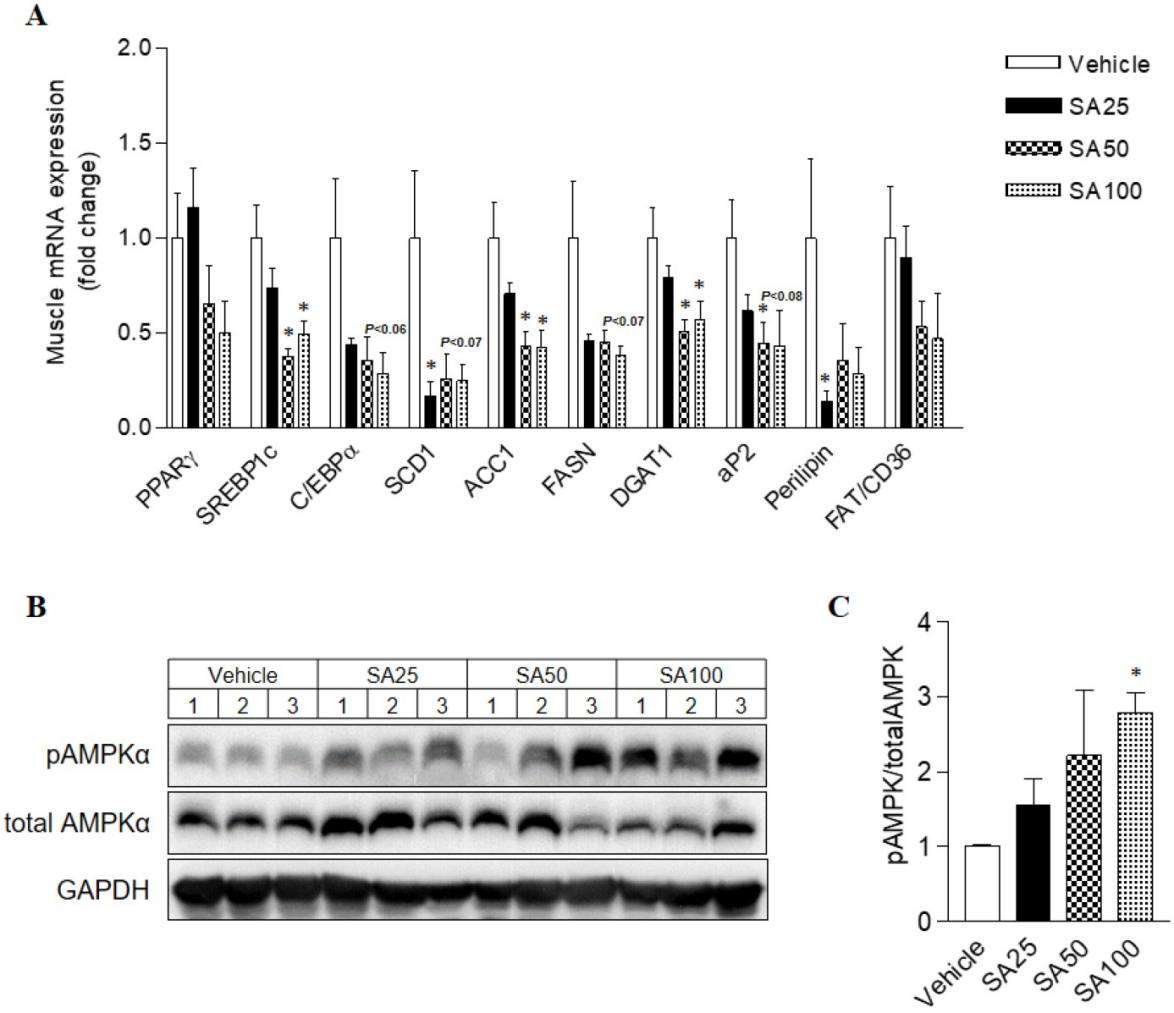
** The effect of SA on the mRNA expression of lipogenic genes and on the protein expression of AMPK in muscle of C57BL/6 mice fed a high-fat diet.** Mice were randomly divided into four groups: high-fat diet (HFD) plus vehicle (0.5% CMC) and HFD plus SA (25, 50, and 100 mg/kg). The mice were fed a HFD only or HFD with SA for 12 weeks. Quantitative PCR analysis of lipogenic genes (A), Representative western blots (B) and band intensity analysis of pAMPK and AMPK (C) measured after 12 weeks of treatment. Values are the mean±SEM (*n* = 5/group). *Significantly different from the HFD-fed vehicle group at *p* < 0.05.

To investigate the molecular mechanism of the anti-obesity effect of SA in WAT, analyses for gene expression related to lipid and energy metabolism were performed in the epididymal fat. The expression of lipogenesis-related genes was mostly decreased in SA-treated groups (**Figure 7A**). Among these genes, expression of peroxisome proliferator-activated gamma (PPARγ), sterol regulatory element-binding protein 1 (SREBP1c), C/EBPα, stearoyl coenzyme A desaturase (SCD1), acetyl CoA carboxylase (ACC1), fatty acid synthase (FASN), diacylglycerol O-acyltransferase 2 (DGAT2), and perilipin was significantly reduced in the SA100 group. On the other hand, PPARα, AMPKα and UCP1, genes related to energy metabolism, expression dramatically increased in the SA-treated group (**Figure 7B**). Similar to the results from WAT, most lipogenesis-related genes expression in muscle of SA groups was reduced (**Figure 8A**). Recent data indicate that AMPK plays a variety role, regulating multiple aspects of whole- body energy balance including appetite, insulin sensitivity and actions of adipokines/cytokines 27. Therefore, pharmacological activation of AMPK could be useful in ameliorating insulin resistance and obesity. We analyzed phosphorylation of AMPK (pAMPK) in muscle and dose-dependently increased protein expression of pAMPK was observed in SA-treated groups (**Figure 8B and C**). As a result, SA exerts anti-obesity effects by decreasing lipogenesis and increasing energy metabolism.

## Discussion

Obesity and associated metabolic syndromes are growing global problems which have taken an epidemic stature over recent decades 28. Obesity, which is defined as the accumulation of body fat, is associated with complications, such as dyslipidemia, insulin resistance, type 2 diabetes, hypertension, and cardiovascular disease 29. This study was carried out to investigate whether SA has anti-obesity effects in a HFD-induced obese mouse model. It was found that SA administration significantly decreased both body weight and fat mass by regulating the expression of genes associated with lipogenesis as well as energy expenditure.

HFD has been shown to cause an elevation of whole-body fat 30, plasma leptin 31 and lipids, including cholesterol levels 32. Elevated body fat and plasma abnormalities are important risk factors for cardiovascular disease 33. Therefore, control over adiposity has been shown to be important in the reduction of cardiovascular disease incidence and prevention of atherosclerosis associated with obesity. This study demonstrated that the SA-treated groups had significantly lower fat weight than the HFD-only group. The results of the histological analysis showed that the increase in adipocyte size induced by a HFD was mitigated by SA treatment. Additionally, SA treatment dose-dependently abolished the increased cholesterol level in the plasma by HFD treatment. Plasma leptin levels generally increase proportionally to fat mass in various obese rodent models 34. Augmented plasma leptin induced by HFD consumption were significantly reduced after SA treatment, suggesting a potential benefits of SA in other metabolic diseases.

Obesity is also characterized as a state of chronic low-grade inflammation of adipocytes resulting in dysregulation in adipokine production and activation of the pro-inflammation pathway 35. As part of the inflammatory process, locally secreted chemokines attract macrophages, which lead to the formation of CLSs. These macrophages release cytokines that perpetuate the inflammatory process and can lead to insulin resistance 36. In this study, more CLSs were detected in the HFD-fed vehicle group, indicating the inflammatory status of adipose tissue after HFD consumption for 12 weeks. The administration of SA significantly decreased the number of CLSs. Very few CLSs were detected in the epididymal fat in SA-treated groups. In addition, increased WAT cytokines (TNFα, IL-6, and IL-1β), F4/80, CCL2, and CCR2 mRNA expression caused by HFD consumption were significantly decreased after SA treatment. IL-6 and IL-1β are proinflammatory cytokines, chronically elevated in the presence of obesity, that affect insulin resistance 37. Consistent with adipose tissue, IL-6 and IL-1β levels in plasma were markedly decreased in SA-treated group. Overall, the data of this study support that SA treatment lowers HFD-induced alterations in visceral adiposity and changes in systemic and local inflammation.

Obesity is associated with an increased risk of developing insulin resistance and type 2 diabetes 38. SA-treated mice exhibited lower fasting blood glucose levels and improved glucose and insulin tolerance than the mice in the HFD-only group. The reduction of body weight after SA treatment may have contributed to the normalization of the fasting blood glucose and insulin levels, which reflected improved insulin secretion and sensitivity evidenced by IP-GTT and IP-ITT. In addition, the HOMA-IR index, which is closely linked to insulin resistance status 39, was significantly increased in HFD-fed mice, but SA treatment substantially reduced the HOMA-IR index.

To study the potential mechanisms underlying weight loss and metabolic improvement after SA administration, the mRNA expression levels of lipid metabolism and energy expenditure-related genes in WAT, which play a vital role in energy metabolism, were investigated. Our previous studies have demonstrated the beneficial effects of SA on hepatic steatosis *in vitro* 18 and *in vivo* 17. The expression levels of key transcription factors regulating lipogenesis, PPARγ, were significantly suppressed in the liver through the administration of SA with a HFD 17. In line with the liver results, PPARγ expression in the WAT significantly decreased through SA treatment. C/EBPα and SREBP1c are key transcription factors involved in adipocyte differentiation, and mature adipocytes express adipocyte-specific genes 40. The mRNA expression of adipocyte-specific genes (SREBP1c, C/EBPα, aP2, FAS, SCD1, and perilipin) were significantly decreased upon SA administration. Furthermore, increased mRNA expressions of energy metabolism-related genes (PPARα, AMPKα) were observed in WAT of SA-treated mice. PPARα and AMPKα activation in adipose tissue inhibits adipogenesis 41 and exerts anti-inflammatory responses 42,43. In addition, SA treatment significantly elevated thermogenic UCP-1 expression. These observations suggest that SA not only inhibits lipogenesis in adipose tissue but also simultaneously increases fatty acid oxidation and energy expenditure, which may contribute to the beneficial effects of SA on adiposity as well as hepatic steatosis. Ectopic lipid deposition, mainly in liver and skeletal muscle, is closely related to the development of insulin resistance and dyslipidaemia 44. Chronic systemic inflammation increased lipogenesis in nonadipose tissues and lipid deposition in nonadipose tissue may trigger ER stress, oxidative stress and apoptosis, thereby causing tissue injuries 45. Also, HFD-induced decrease of AMPK expression and activity in muscle was associated with systemic insulin resistance and hyperleptinemia 46. In our experiment, SA treatment down-regulated muscle lipogenic gene expression whereas pAMPK protein expression was considerably increased in dose-dependent manner, suggesting AMPK activation in WAT and muscle may be another action of SA in improving insulin sensitivity in a HFD-induced insulin resistance model.

Taken together, SA relieves metabolic disorders by inhibiting body weight increases, reducing fasting blood glucose levels, and ameliorating insulin tolerance in HFD-induced obese mouse models. Moreover, the anti-obesity effects of SA occur through the control of adipogenesis and energy metabolism in adipose tissue and muscle, suggesting that SA might be a potential candidate to prevent or treat obesity and its associated complications.

## Figures and Tables

**Table 1:** Primer sequences used for RT-qPCR

Gene	Primer sequence
**ACC1**	Forward: TGATGCAGAGGTACC
	Reverse: CGTAGTGGCCGTTCT
**AMPKα**	Forward: AACATGGGCGGGTTGAAGA
	Reverse: ATCCACGGCAGACAGGATCT
**AMPKβ**	Forward: AGGACACGGGCATCTCTTGT
	Reverse: TGGTTCAGCATGACGTGGTT
**aP2**	Forward: GGCCAAGCCCAACATGATC
	Reverse: CACGCCCAGTTTGAAGGAAA
**ATGL**	Forward: CTGTCTTGCGCCACCTACAG
	Reverse: GCTGACGCTGGCATTCTTC
**C/EBPα**	Forward: CGCAAGAGCCGAGATAAAGC
	Reverse: CACGGCTCAGCTGTTCCA
**CCL2**	Forward: CAGCAAGATGATCCCAATGAGTAG
	Reverse: TCTCTTGAGCTTGGTGACAAAAAC
**CCR2**	Forward: GGGCTGTGAGGCTCATCTTT
	Reverse: TGCATGGCCTGGTCTAAGTG
**DGAT1**	Forward: TTTGTTGTGGCTGCATTTCAG
	Reverse: TGATTGTGGCCAGGTTAACCA
**DGAT2**	Forward: GTGGCCTGCAGTGTCATCCT
	Reverse: TGGGCGTGTTCCAGTCAAAT
**F4/80**	Forward: GATGAATTCCCGTGTTGTTG
	Reverse: ACATCAGTGTTCCAGGAGAC
**FASN**	Forward: GATCCTGGAACGAGAACACGAT
	Reverse: GAGACGTGTCACTCCTGGACTTG
**FAT/CD36**	Forward: GAGCCTTCACTGTCTGTTGGAA
	Reverse: CTGCTACAGCCAGATTCAGAACTG
**Perilipin**	Forward: TGGAGAGTAAGGATGTCAATGAACA
	Reverse: CCACAGGCAGCTGCAGAAC
**PPARα**	Forward: TGGCAAAAGGCAAGGAGAAG
	Reverse: CCCTCTACATAGAACTGCAAGGTTT
**PPARγ**	Forward: CCACTCGCATTCCTTTGACA
	Reverse: TGGGTCAGCTCTTGTGAATGG
**SCD1**	Forward: GCCTCTGGAGCCACAGAACT
	Reverse: GCCCATTCGTACACGTCATTC
**SREBP1c**	Forward: GGCCGAGATGTGCGAACT
	Reverse: CCCGGGAAGTCACTGTCTTG
**UCP1**	Forward: CCCTGGCAAAAACAGAAGGA
	Reverse: AGCTGATTTGCCTCTGAATGC
**18s**	Forward: GACACGGACAGGATTGACAGATTGATAG
	Reverse: GTTAGCATGCCAGAGTCTCGTTCGTT

## Abbreviations

SA: *Sicyos angulatus*
HFD: high-fat diet
IL: interleukin
TNFα: tumor necrosis factor-α
KFSC: Korean Food Standard Codex
RT: room temperature
H&E: hematoxylin and eosin
CLS: Crown-like structure
IP-GTT: intraperitoneal glucose tolerance test
IP-ITT: intraperitoneal insulin tolerance test
AUC: area under the curve
HOMA-IR: homeostatic index of insulin resistance
RT-qPCR: Reverse transcription-quantitative polymerase chain reaction
PPARγ: peroxisome proliferator-activated gamma
SREPB1c: sterol regulatory element-binding protein 1
ACC1: acetyl CoA carboxylase
SCD1: stearoyl coenzyme A desaturase
FASN: fatty acid synthase
DGAT: diacylglycerol O-acyltransferase
